# Examining the effects of adjuvant chemotherapy on cognition and the impact of any cognitive impairment on quality of life in colorectal cancer patients: study protocol

**DOI:** 10.1186/s40359-015-0100-5

**Published:** 2015-11-26

**Authors:** Marie-Rose Dwek, Lorna Rixon, Alice Simon, Catherine Hurt, Stanton Newman

**Affiliations:** School of Health Sciences, City University London, 10 Northampton Square, London, EC1V 0HB UK

**Keywords:** Cognitive dysfunction, Chemotherapy, Colorectal cancer, Quality of life

## Abstract

**Background:**

Research suggests that chemotherapy can cause deficits in both patients’ objectively measured and self-reported cognitive abilities which can in turn affect their quality of life (QoL). The majority of research studies have used post-treatment retrospective designs or have not included a control group in prospective cohorts. This has limited the conclusions that can be drawn from the results. There have also been a disproportionate number of studies focussed on women with breast cancer, which has limited the generalisability of the results to other cancer populations.

**Aim:**

This study aims to identify the extent and impact of chemotherapy-induced cognitive decline in colorectal cancer patients. Possible associations with poorer QoL will also be explored.

**Design:**

This will be a longitudinal controlled cohort study. Questionnaires measuring subjective cognitive functioning, QoL, fatigue and mood, and neuropsychological assessments of objective cognitive function will be collected pre-, mid- and post- chemotherapy treatment from a consecutive sample of 78 colorectal cancer patients from five London NHS Trusts. A further 78 colorectal cancer surgery only patients will be assessed at equivalent time points; this will allow the researchers to compare the results of patients undergoing surgery, but not chemotherapy against those receiving both treatments.

Pre- and post-chemotherapy difference scores will be calculated to detect subtle changes in cognitive function as measured by the objective neuropsychological assessments and the self-reported questionnaires. A standardised z-score will be computed for every patient on each neuropsychological test, and for each test at each time point. The post-chemotherapy score will then be subtracted from the pre-chemotherapy score to produce a relative difference score for each patient.

ANCOVA will be used to compare mean difference z-scores between the chemotherapy and surgery-only groups while controlling for the effects of gender, age, depression, anxiety, fatigue and education.

**Discussion:**

The result from this study will indicate whether a decline in cognitive functioning can be attributed to chemotherapy or to disease, surgical or some other confounding factor. Identification of risk factors for cognitive deficits may be used to inform targeted interventions, in order to improve QoL and help patients’ cope.

## Background

Chemotherapy has been shown to increase survival for a range of different cancers. This impact has improved considerably over the years - most notably for breast cancer and colorectal cancer (CRC) [1]. However, these drugs can cause severe side effects; the most commonly perceived amongst the general public have changed in recent years from nausea, vomiting, loss of appetite and hair loss [2] to fatigue and psychosocial QoL concerns [3]. This is due the fact that there has been a significant reduction in chemotherapy-associated toxicities [3] and the use of very effective anti-nausea medications.

Many cancer patients also report a decline in cognitive function following chemotherapy, colloquially referred to as “chemofog” or “chemobrain”. Research in this area suggests that memory, processing speed and executive function may decline as a result of chemotherapy treatments following surgery [4–9]. These cognitive deficits could have implications for patients’ QoL, daily functioning and work activity for long term cancer survivors and are therefore an important concern [10]. The natural course and extent of any cognitive decline over time and whether this decline translates into observable functional difficulty for patients is relatively unknown.

“Chemobrain” was first identified by female breast cancer survivors [11]. The majority of existing research has taken place in this patient group [12, 13] where a number of differing treatment combinations (e.g. anaesthetics and hormonal therapies) could augment the effects on cognitive dysfunction. Chemotherapy induced cognitive impairment research has probably continued to focus on the female breast cancer population because it is the most frequently diagnosed cancer in women in the world, comprising 18 % of all female cancers [11]; with 78 % of those diagnosed surviving for ten or more years [1]. However, this focus has precluded the possibility of exploring gender differences in cognitive decline, despite reports from other, mixed-gender, cancer populations (such as lung and CRC patients) that both men and women are affected by the same constellation of symptoms [11, 14].

CRC patients are an obvious population in which to carry out this type of research in a mixed-gender setting. CRC is the fourth most prevalent cancer in many developed countries, affecting men and women almost equally [15]. Such patients have a comparatively high survival rate. After surgery, 48 % of those with Stage three bowel cancer will live for at least 5 years [16]. The majority of resected Stage three CRC patients are offered a 24-week course of adjuvant chemotherapy, administered as part of standard treatment. This makes them a good patient group for a longitudinal study examining chemotherapy-induced cognitive changes over time.

One of the major limitations of earlier research has been a failure to measure cognitive function prior to chemotherapy treatment in order to provide a baseline against which to detect changes over time and to determine whether there was impairment prior to the commencement of the treatment [12, 17]. Measuring cognitive function both before and after chemotherapy treatment would identify any changes occurring due to treatment.

The few recent longitudinal studies have produced mixed findings [18–22]. One study reported a subtle negative influence of chemotherapy on cognitive function in breast cancer patients compared to women receiving only adjuvant hormonal therapy [21]. One of the few longitudinal studies in CRC patients reported no effect on cognitive function [22]. This study, however, used only a small sample (*N* = 57)), whilst Cruzado and colleagues (2014) [14] found that at the end of adjuvant chemotherapy treatment for CRC there was an acute decline in verbal memory in 56 % of patients. Neither of these two studies used a control group which meant it was not possible to establish whether any differences in cognitive functioning were due to the general effects of cancer and its symptoms or to the chemotherapy treatment. Altogether this represents a limited research base with a limited methodology and as a result it is not possible to draw firm conclusions about the extent and nature of any cognitive decline arising from chemotherapy treatment.

Our proposed study will address the limited generalisability of the existing literature by examining a larger CRC population, assessing patients pre-, mid- and post-treatment. The design also follows one of the International Cognition and Cancer Task Force (ICCTF) recommendations to compare “*patients who receive the same ensemble of treatments with or without chemotherapy”* [18] by including a surgery only control group (www.icctf.com) [18].

All of the participants in this study will have undergone the same type of surgery but those in the control group will not go on to have chemotherapy treatment. However, it is expected that all participants will experience the same range of emotions such as anxiety and depression that can accompany a cancer diagnosis and consequent treatment. This research design will allow the research team to detect the effect that adjuvant chemotherapy may have on cognition in addition to surgery. It will also permit the researchers to control for the impact of a cancer diagnosis on levels of psychological distress and QoL, both of which might affect cognitive functioning. Although it is recognised that cancer severity may differ between the chemotherapy and surgery only groups, these effects will be controlled for statistically. It would not be feasible to attempt to match participants for disease severity because those who are offered adjuvant chemotherapy following surgery would usually have a more advanced cancer stage than those who require no further treatment after surgery.

The ICCTF also developed recommendations for a core set of neuropsychological tests, common criteria for defining cognitive impairment and cognitive changes, and common approaches to study methods across such research [18]. These will be followed in this study.

### Study objectives

The aim of this study is to establish the extent of chemotherapy-induced cognitive deficits and its effect on QoL and daily functioning in both men and women undergoing treatment for CRC. Specifically the study will:

1. Determine the extent of cognitive deficits attributable to adjuvant chemotherapy treatment by conducting neuropsychological assessments in CRC patients pre-, mid- and post-treatment.
2. Compare the extent and pattern of cognitive deficits in CRC patients against a similar control group (e.g. patients who have had colorectal surgery but do not require chemotherapy).
3. Determine the effect of treatment-related cognitive decline on QoL and psychological distress.
4. Examine the relationship between patients’ self-reported cognitive functions and their objectively assessed cognitive functions.

## Methods

### Design

**Fig. 1:**
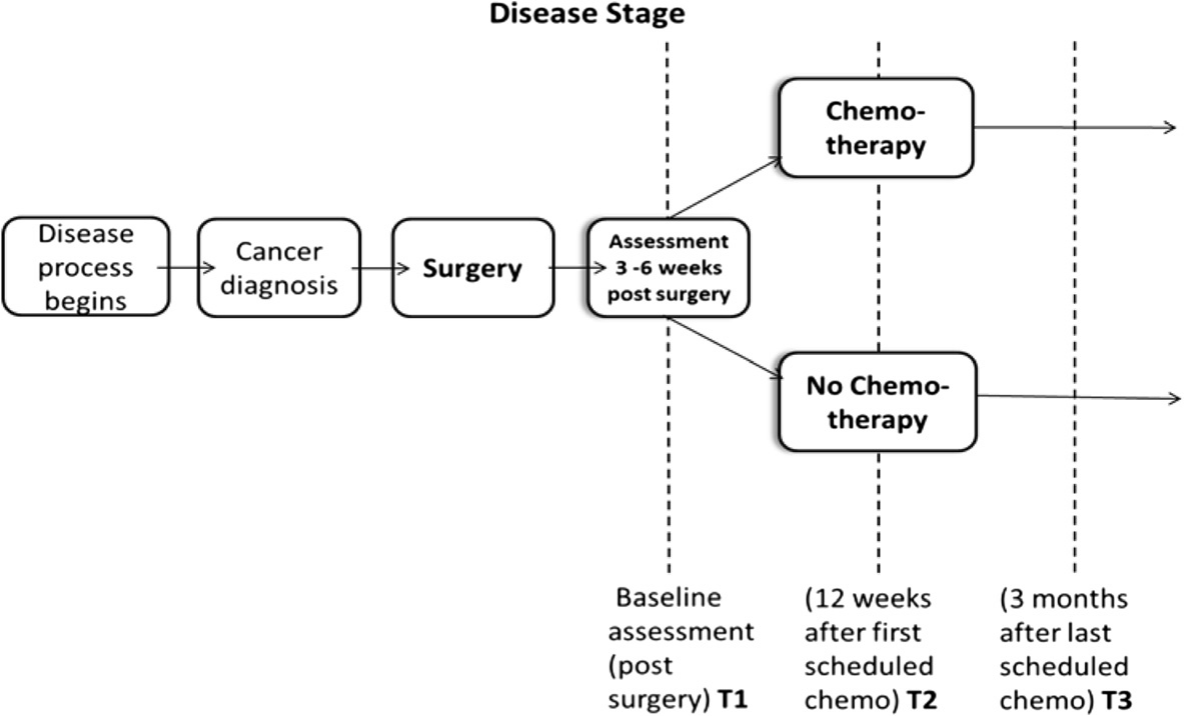
Study measurement time points

This study will use a longitudinal design. Data will be collected using neuropsychological assessments and QoL questionnaires at three time points: post-surgery but prior to chemotherapy treatment (‘T1’), between 12 and 14 weeks after first scheduled chemotherapy (‘T2’), and 3 months after last scheduled chemotherapy (‘T3’) (Please see Fig. 1). A total of 156 participants (50 % of whom will receive chemotherapy and 50 % will be non-chemotherapy surgical patients) will be recruited from 5 NHS Healthcare Trusts across London. For those patients who are not receiving chemotherapy data will be collected at T1 and then in parallel with the chemotherapy group at T2 and T3.

### Participants

Adult CRC patients under the care of the Consultant Oncologists at five participating NHS Trusts with London-based hospital sites who have had colorectal surgery will be invited to take part in the study.

Patients who have had prior exposure to chemotherapy or significant psychiatric or medical comorbidities, which might affect ability to participate in the study, will be excluded. Patients who are not sufficiently literate in English will also be excluded as a failure to understand English would make completion of the questionnaires impossible. Only those patients over the age of 18 years who have had surgery following a diagnosis of CRC will be eligible to participate, provided that they are then offered adjuvant chemotherapy treatment and start it at least 3 weeks after surgery or no other cancer related treatments at all and are fluent in written and spoken English.

During the post-surgery follow-up appointment nurses/trial co-ordinators at each location will provide a consecutive series of patients (satisfying the inclusion criteria) with information about the study. A research assistant will also be available at that time to answer any questions that the patient may have about the study. Those patients who provide the researcher with telephone numbers will be contacted after 48 h and invited to participate in an interview, either at their chemotherapy clinic or at home. This interview will take place prior to the commencement of chemotherapy treatment for those in receipt of chemotherapy and at a parallel point in time for the surgery only control group (T1). At the beginning of the interview the patient will be guided through the information sheet again and the consent form by the researcher and written informed consent will be obtained. In the event that a patient declines to provide the researcher with a telephone number or refuses to take part he/she will not be contacted again about the study.

The questionnaires and assessments will be completed by the patient in the hospital at T1 and an appointment for the subsequent assessments (T2, T3) will be made. Patients’ participation in the research will take approximately 2 h and 15 min at T1 and 1 h 50 min at T2 and T3 and will take place at the time of the appropriate outpatient appointment or at an equivalent point in time for the control group.

### Measures

#### Pre-screening test

At T1, consented participants over the age of 65 will be asked to complete the Montreal Cognitive Assessment (MoCA) version 3 [23] as a pre-screening test in order to exclude those with mild cognitive impairment (MCI) from taking part in the study. In the event that such a participant obtains a raw score of less than 26 they will not progress into the study, as this is considered to be the cut off point for MCI.

The following measures will be collected at T1, T2 and T3 unless otherwise specified:

#### Neuropsychological assessments

The following battery of assessments has been designed to measure a wide range of cognitive domains and includes all of those recommended by the ICCTF [10]. All measures are standardised, validated and taken from published test batteries with healthy population norms, which will provide the researchers with another important comparison:

i) The Hopkins Verbal Learning Test-Revised (HVLT-R) [24] for verbal memory; this is a brief verbal learning and memory test that includes delayed recall and recognition trials. Alternate forms will be used at each of T1, T2 and T3.
ii) Trail Making Test (TMT) A and B [25] to measure psychomotor speed and aspects of executive function and spatial organisation, visual pursuits, recall, and recognition.
iii) The Controlled Oral Word Association (COWA) of the Multilingual Aphasia Examination [26] that measures speeded lexical fluency requiring aspects of executive function.

The above-recommended measures will be supplemented with the following:

iv) The Digit Span subtest of the Wechsler Adult Intelligence Scales – Third Edition, (WAIS - III Digit Span) [27] consisting of two mental activity tests involving auditory attention and short term memory retention capacity.
v) The Symbol Digit Modalities Test (SDMT) [28, 29] assesses complex visual scanning and tracking [29]. It is a simple substitution task.
vi) Letter Cancellation of the Behavioural Inattention Test (BIT) [30, 31].
vii) Grooved Pegboard Test [32, 33], a manual dexterity test measuring visuo-motor coordination.
viii) The Benton Visual Retention Test (BVRT) [34] for visual perception, visual memory and visuo-constructive ability. There are three near-equivalent forms (Forms C, D, and E) of the BVRT. Form C will be used at T1, Form D at T2 and Form E at T3, which will allow for retesting while minimizing practice effects. Administration A (of the 4 possible methods) will be used throughout.

#### Self-reported cognitive functioning

Functional Assessment of Cancer Therapy-Cognitive scale (FACT-Cog, Version 3) [35] is a validated self-report measure of cognitive function. It evaluates mental acuity, attention and concentration, memory, verbal fluency, functional interference, deficits observed by others but reported by the patient; change from previous functioning, and impact on quality of life.

#### Mood

Anxiety and depression will be measured using the Hospital Anxiety and Depression Scale (HADS) [36].

#### Fatigue

The Functional Assessment of Chronic Illness Therapy – Fatigue (FACIT-F, version 4) [37] a 13-item self-report subscale of the FACT-G (see below). The FACIT-F is a well-validated quality of life instrument widely used for the assessment of cancer-related fatigue in clinical trials [37–40]. The items include physical and functional consequences of fatigue [37].

#### Quality of life

The Functional Assessment of Cancer Therapy - General (FACT-G, Version 4), will be used to measure 4 quality of life domains [38]: physical, emotional, family/social and functional well being in the previous 7 days. Participants will also complete the 9-item FACT-C subscale that evaluates symptoms related specifically to CRC.

#### IQ

This will only be measured at T1 using the Wechsler Abbreviated Scale of Intelligence – Second Edition (WASI –II) Vocabulary and Matrix Reasoning [41] to assess background level of intellectual ability.

#### Socio-demographic information

This information will be collected at T1 via a structured questionnaire and will include age, sex, employment (i.e. full or part-time employment, retired and unemployed) and marital status (married, cohabiting, single, separated, divorced, widowed). Specific information relating to surgery date, planned treatments and comorbidities will also be obtained.

#### Medical records and treatment plan

Participants’ disease and treatment-related factors will be recorded from medical records (including the type of chemotherapy administered, any dose adjustments made, the actual number of cycles completed, any neurotoxicity experienced and the anti-emetic regimen) once the participant has consented.

#### Sample size

A recent meta-analysis of chemotherapy and cognitive function [42] estimated mean effect sizes in a range of cognitive domains. The effect sizes ranged from d =−0.11 to−0.51. A sample size calculation was performed using GPower 3.1. Taking into consideration the resource constraints of the study, the sample size was calculated with the aim of detecting a medium effect size. To detect an effect size of−0.26 with 80 % power and a significance level of 0.05 at the final time point, a minimum sample size of 120 participants was indicated. Based on medium effect sizes in the meta-analysis, a sample size of 120 would allow effects to be detected in the following domains: executive function, information processing speed, language, motor function, verbal memory and visual memory. However, it is acknowledged small effects may not be detected in the following domains: attention and visuospatial skills. Assuming an overall attrition rate of 22 % (based on SCOT trial attrition rates^1^), a total sample size of 156 participants will be sought (78 per group).

#### Analysis

In order to detect subtle changes in cognitive function, pre- and post-chemotherapy difference scores will be calculated. This approach has been successfully applied in cardiac research exploring post-surgery cognitive decline [43]. A standardised score (z-score) will be computed for every patient on each neuropsychological test by dividing the test score by the standard deviation of the pre-chemotherapy test score of all study participants. A standardised score will be computed for each test at all-time points using the pre-chemotherapy standard deviation. The post-chemotherapy standardised score will then be subtracted from the pre-chemotherapy standardised score to give a relative difference score for each patient. A total z-score can then be computed for all neuropsychological tests.

ANCOVA will be used to compare mean difference z-scores between the chemotherapy and surgery-only groups while controlling for the effects of gender, age, depression, educational level and extent of disease. This method of analysis is preferable to conventional deficit/no deficit analysis as it allows for detection of subtle changes in cognition and accounts for pre-chemotherapy cognitive performance [44] and will increase the power of the analyses.

Multiple and logistic regression analyses will be used, as appropriate, to explore the relationship between cognitive impairment (total z-score) and quality of life (FACT G & C), adjusting for age, gender, SES and anxiety and depression (HADS). Finally, correlation and regression analyses will be also used to initially examine the relationship between subjective (FACT-Cog) and objective (total z-score) cognitive impairment.

### Ethics and acceptability feasibility

Ethical approval for the study was obtained from the NHS Health Research Authority – NRES Committee South-West Cornwall & Plymouth in August 2013.

All of the assessments are standardised and have been widely used across many patient groups including cancer patients. At the beginning of each assessment participants will be reminded that they have the right to withdraw at any time and can avoid answering questions that are felt to be too personal or intrusive. Participants will be assured that any future treatment will not be affected in any way should they choose to withdraw. However, in the unlikely event that the assessments and content of the questionnaires cause distress or any discomfort to any of the participants, the researcher will remind the participant that he/she is entitled to refuse to answer any question that may cause upset or distress and that he/she may stop and withdraw from the study at any time. If they feel the need to have professional help they will be encouraged to raise this with their consultant or the consultant will be informed by the researcher if the patient would prefer.

#### Data management and data confidentiality

Confidentiality will be adhered to at all times. All questionnaires will be kept anonymous by assigning codes to participants. All data will only be identified by that code, not by the participant name or any other information that could identify them. All questionnaires will be kept in locked cabinets and/or password protected computers.

Data will be collected, transferred and stored in compliance with the NHS data protection requirements and be managed by a data manager. The data manager will also advise on current regulatory framework regarding data protection and data management procedures in compliance with the Data Protection Act 1998 and other regulations. The data manager will design and set up a bespoke database in MS Access, which will have integrated data validation checks and a full audit trail. Patient identifiable and pseudonymised data will be stored separately. The data manager will advise on and set up data transfer systems and encryption systems so that all patient identifiable data is encrypted. The data manager will also advise on storage, back up and archiving of data to ensure databases are regularly backed up to ensure data is safeguarded from accidental loss. The study master file and all study documentation will be archived for 10 years.

## Discussion

At the time of writing, a feasibility study based on this protocol is being carried out to assess participant numbers, attrition rates, recruitment procedures and methodology.

The proposed study has a number of strengths. It is a multi-site study that should provide access to large numbers of potential participants, thus ensuring that the findings are more generalizable than results garnered from a single site study. In contrast to other studies in this area, this project is longitudinal and includes a comparison group in a gender-neutral cancer population. This follows advice supplied by the ICCTF. The study also uses all of the core neuropsychological tests recommended by the ICCTF.

The study includes a pre-screening tool in order to exclude anyone with pre-existing cognitive dysfunction from taking part. This has been done in few studies to date, leaving open the possibility that the results could be skewed by those who have pre-existing cognitive conditions. The MoCA was specifically designed as a rapid screening instrument for MCI. It assesses different cognitive domains relative to our study in 10 min, which should allow us to preclude those potential participants with existing cognitive deficits.

The study does also have some limitations. Treatment regimes differ across participants such that some are prescribed intravenous treatments every 2 weeks whilst others take oral tablets every 3 weeks. Additionally, treatment regimes can change over time for some patients, and this may mean that the treatment protocol will not be the same for all chemotherapy patients. Secondary analysis will be conducted if noticeable differences are identified between chemotherapy regimens although these results will need to be interpreted with caution in the event that this does occur as the comparisons will be underpowered.

It is also acknowledged that the time from diagnosis to start of treatment (particularly the period between the surgery and the start of chemotherapy) is an emotionally stressful time. Given that the anaesthetic from surgery and general emotional distress can have an adverse effect on cognitive functioning; it may be that testing during this period will not provide a true index of abilities [45]. To control for this the measures of emotional distress will be examined in relation to cognitive performance. However as per the ICCTF’s recommendations, given the logistical difficulties of carrying out the assessments pre surgery, they are all being done after surgery but before adjuvant chemotherapy treatment begins.

### Potential research implications

The result from this study will indicate whether a decline in cognitive functioning can be attributed to chemotherapy or to disease, surgical or some other confounding factor. Identification of risk factors for cognitive deficits may be used to inform targeted interventions, either compensatory or rehabilitating cognitive strategies to manage cognitive deficits or challenging unhelpful perceptions of cognitive functioning to lessen the negative effects on QoL.

### Potential benefits to research participants

There are no immediate benefits for the research participants. There is anecdotal evidence to suggest that cancer patients like to talk about ‘chemofog/chemobrain’ so there is a small benefit in terms of validation that such participants may feel by being asked about this effect; however this may only be apparent in their subjective views of their cognition.

There will be long term benefits to future cancer patients in terms of the possibility of making a direct contribution to the improvement of cancer patients’ lives that may ultimately lead to changes in care. For example, results may lead to making the case for integrating neuropsychological assessments into the treatment programme in order to identify those with specific deficits and unfulfilled needs.

## Abbreviations

CRC: Colorectal cancer
QoL: Quality of life
T1: Post-surgery, prior to chemotherapy treatment (i.e. approx. 3–6 weeks after surgery)
T2: Twelve weeks after first scheduled chemotherapy or a parallel point in time
T3: Three months after last scheduled chemotherapy (i.e. approx. 9 months after T1)
MoCA: Montreal cognitive assessment version 3
HVLT-R: The hopkins verbal learning test-revised
TMT: Trail making test
COWA: The controlled oral word association
SDMT: The symbol digit modalities test
BIT: Letter cancellation of the behavioural inattention test
BVRT: The benton visual retention test
FACT – Cog: Functional assessment of cancer therapy-cognitive scale
HADS: Hospital anxiety and depression scale
FACIT –F: The functional assessment of chronic illness therapy –fatigue version 4
FACT – G: The functional assessment of cancer therapy - general version 4
WASI-II: Wechsler abbreviated scale of intelligence – second edition vocabulary and matrix reasoning

## Footnotes

1 Personal communication with Dr Bridgewater of UCLH.
